# Associations between plasma branched-chain amino acids, β-aminoisobutyric acid and body composition

**DOI:** 10.1017/jns.2015.37

**Published:** 2016-02-03

**Authors:** Annemarie Rietman, Takara L. Stanley, Clary Clish, Vamsi Mootha, Marco Mensink, Steven K. Grinspoon, Hideo Makimura

**Affiliations:** 1 Division of Human Nutrition, Wageningen University, NL-6703 HD Wageningen, The Netherlands; 2 Program in Nutritional Metabolism and Neuroendocrine Unit, Massachusetts General Hospital and Harvard Medical School, Boston, MA 02114, USA; 3 Pediatric Endocrine Unit, Massachusetts General Hospital, Boston, MA 02114, USA; 4 Broad Institute of MIT and Harvard, Cambridge, MA 02142, USA; 5 Center for Human Genetic Research, Massachusetts General Hospital and Harvard Medical School, Boston, MA 02114, USA

**Keywords:** Branched-chain amino acids, Visceral adiposity, β-Aminoisobutyric acid, Subcutaneous adipose tissue, Lean body mass, Metabolomics, AU, arbitrary units, B-AIBA, β-aminoisobutyric acid, BCAA, branched-chain amino acid, BCAT, branched-chain amino acid aminotransferase, BCKD, branched-chain α-ketoacid dehydrogenase, DXA, dual-energy X-ray absorptiometry, HOMA-IR, homeostasis model assessment for insulin resistance, OGTT, oral glucose tolerance test, SAT, subcutaneous adipose tissue, VAT, visceral adipose tissue

## Abstract

Plasma branched-chain amino acids (BCAA) are elevated in obesity and associated with increased cardiometabolic risk. β-Aminoisobutyric acid (B-AIBA), a recently identified small molecule metabolite, is associated with decreased cardiometabolic risk. Therefore, we investigated the association of BCAA and B-AIBA with each other and with detailed body composition parameters, including abdominal visceral adipose tissue (VAT) and subcutaneous adipose tissue (SAT). A cross-sectional study was carried out with lean (*n* 15) and obese (*n* 33) men and women. Detailed metabolic evaluations, including measures of body composition, insulin sensitivity and plasma metabolomics were completed. Plasma BCAA were higher (1·6 (se 0·08) (×10^7^) *v.* 1·3 (se 0·06) (×10^7^) arbitrary units; *P* = 0·005) in obese *v.* lean subjects. BCAA were positively associated with VAT (*R* 0·49; *P* = 0·0006) and trended to an association with SAT (*R* 0·29; *P* = 0·052). The association between BCAA and VAT, but not SAT, remained significant after controlling for age, sex and race on multivariate modelling (*P* < 0·05). BCAA were also associated with parameters of insulin sensitivity (Matsuda index: *R* −0·50, *P* = 0·0004; glucose AUC: *R* 0·53, *P* < 0·001). BCAA were not associated with B-AIBA (*R* −0·04; *P* = 0·79). B-AIBA was negatively associated with SAT (*R* −0·37; *P* = 0·01) but only trended to an association with VAT (*R* 0·27; *P* = 0·07). However, neither relationship remained significant after multivariate modelling (*P* > 0·05). Plasma B-AIBA was associated with parameters of insulin sensitivity (Matsuda index *R* 0·36, *P* = 0·01; glucose AUC: *R* −0·30, *P* = 0·04). Plasma BCAA levels were positively correlated with VAT and markers of insulin resistance. The results suggest a possible complex role of adipose tissue in BCAA homeostasis and insulin resistance.

Worldwide, the number of people suffering from obesity continues to increase^(^^1^^)^. Obesity is characterised by increases in both the abdominal subcutaneous adipose tissue (SAT) and visceral adipose tissue (VAT). SAT is considered to have more protective properties in relation to cardiometabolic risk factors^(^^2^^)^, whereas VAT is detrimental to cardiometabolic health^(^^3^^,^^4^^)^.

Obesity is also associated with elevated levels of plasma branched-chain amino acids (BCAA)^(^^5^^)^. The BCAA valine, leucine and isoleucine are associated with insulin resistance^(^^6^^–^^11^^)^. Acute increases in plasma amino acids worsen insulin sensitivity^(^^12^^)^, and higher levels of dietary protein intake is associated with impaired glucose metabolism^(^^13^^)^. Furthermore, levels of baseline circulating BCAA predicted the development of incident diabetes in a large longitudinal cohort study^(^^11^^)^ suggesting that the relationship between BCAA and insulin resistance may be causal. However, the relationship between BCAA and detailed parameters of body composition, specifically VAT, has yet to be reported.

β-Aminoisobutyric acid (B-AIBA), a small molecule metabolite, was recently identified using a metabolomics approach as a possible novel myokine that increases browning of white adipocytes in response to physical activity and was found to be inversely associated with cardiometabolic risk including fasting glucose, insulin and homeostasis model assessment (HOMA) in addition to TAG and cholesterol^(^^14^^)^. As B-AIBA can be formed by the catabolism of thymine and valine^(^^14^^,^^15^^)^ this may represent a possible pathway through which BCAA exert their metabolic effects. The catabolism of BCAA, valine in particular, could decrease circulating BCAA while increasing B-AIBA, both of which are associated with improved insulin resistance.

In the present study we investigated the association of BCAA and B-AIBA with each other and with detailed body composition parameters, including SAT and VAT, for the first time. We hypothesised that BCAA would be positively associated with VAT while B-AIBA would be negatively associated with VAT. This hypothesis was evaluated in a cross-sectional study of lean and obese men and women for whom detailed metabolic evaluations were performed.

## Materials and methods

### Subjects

A total of forty-eight lean (BMI < 25 kg/m^2^; *n* 15) and obese (BMI ≥ 30 kg/m^2^; *n* 33) men and women from the Boston community were evaluated between November 2007 and March 2009 at the Massachusetts Institute of Technology and Massachusetts General Hospital Clinical Research Center. Subjects were between the ages of 18 and 55 years and were otherwise healthy. Subjects receiving anabolic steroids, glucocorticoids, testosterone, hormone replacement, hormonal contraception, growth hormone or medication for diabetes mellitus treatment were excluded. Subjects with a Hb level less than 110 g/l, creatinine above 15 mg/l, aspartate aminotransferase more than 2·5-fold above the upper limit of normal, and chronic illness such as HIV were also excluded. Written informed consent was obtained from each subject before testing in accordance with the Committee on the Use of Humans as Experimental Subjects of the Massachusetts Institute of Technology and the Subcommittee on Human Studies at the Massachusetts General Hospital.

### Body composition analyses

Anthropometric measurements including height, body weight, and waist and hip circumference were obtained in triplicate by a trained nutritionist after an overnight fast. Total body fat percentage was determined by dual-energy X-ray absorptiometry (DXA) testing using a Hologic-4500 densitometer (Hologic, Inc.). DXA uses a three-compartment model, partitioning tissue into lean, bone and fat mass. We performed 1 cm cross-sectional abdominal computed tomography (CT) scans at the level of L4 to assess the distribution of abdominal SAT and abdominal VAT as previously described^(^^16^^)^.

### Biochemical analyses

Fasting blood samples were drawn and oral glucose tolerance tests (OGTT) were performed using the 75 g oral glucose challenge. Glucose and insulin were obtained at 0, 30, 60, 90 and 120 min. Homeostasis model assessment for insulin resistance (HOMA-IR) and the Matsuda index^(^^17^^)^ were calculated using the following equations: 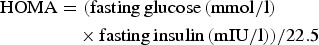


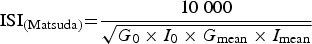

*G*_mean_ and *I*_mean_ were obtained from values at time points 0, 30, 60, 90 and 120 min.

Measurement of fasting cholesterol profile was performed on a separate visit. Glucose and lipid levels were determined using standard methodology in the Massachusetts Institute of Technology clinical laboratory. Insulin was measured by a paramagnetic-particle chemiluminescence immunoassay using the Beckman Access Immunoassay System (Beckman Coulter). The analytical sensitivity of the assay is 0·03 IU/ml, and the precision is 3–5·6 %.

### Metabolomic profiling

Metabolomic profiling was performed using liquid chromatography tandem mass spectrometry (LC-MS) for sixty-five polar metabolites including BCAA and B-AIBA from fasting plasma samples^(^^11^^,^^18^^)^. Polar metabolites were analysed in the hydrophilic interaction liquid chromatography/negative ion MS mode using targeted multiple reaction monitoring MS scans for optimal analytical sensitivity. To create this targeted profiling method, declustering potentials and collision energies were optimised for each metabolite by infusion of reference standards. In this method, the range of analyte signals spans at least four orders of magnitude dynamic range. Results are output in arbitrary units (AU). In general, the CV for repeated analyses are inversely proportional to the magnitude of the instrument response. Median CV was determined using repeated analyses of a pooled plasma reference sample (*r* 10), and was 4·3 %; >70 % of metabolites had a CV ≤ 10 %. The BCAA leucine, isoleucine and valine were evaluated individually and summed and evaluated as total BCAA.

### Dietary evaluation

Absolute intake of macronutrients including carbohydrates, proteins and fat were assessed by collection of a 4-d food record facilitated by a trained research dietitian during direct interview. Data were analysed using Nutrition Database Systems for Research (NDSR) software with the NDSR 2008 (version 2; University of Minnesota, Minneapolis, MN).

### Statistical analyses

All data are expressed as mean values with their standard errors. Normality of distribution was assessed using Shapiro–Wilk analyses. Parameters that were not normally distributed were log transformed before analyses. All metabolites from the metabolomics assay were log transformed before analyses. A targeted statistical analysis using Pearson univariate regression analysis was performed to determine the relationship of (individual) BCAA and B-AIBA with body composition and metabolic parameters. As a secondary analysis, a non-targeted metabolomics analysis was performed assessing the relationship of all sixty-five polar metabolites to various body composition parameters. Multivariate regression analysis was performed evaluating metabolomic parameters and body composition parameters that were significant on univariate analysis. For body composition parameters, the model controlled for age, sex and race. Additionally, to investigate whether a relationship between (individual) BCAA and VAT can be explained by insulin resistance, the Matsuda index was included in a multivariate model. For the metabolic parameters, the model included age, sex, race and BMI. For our primary end point assessing the relationship of (individual) BCAA with body composition parameters, *P* values were considered significant if *P* < 0·05, as this was a targeted analysis focusing specifically on (individual) BCAA. For the non-targeted secondary analyses evaluating all metabolites, *P* values were considered significant if <0·00074 for Bonferroni correction for multiple comparisons.

Statistical analyses were performed with SAS 9.2 (2002–2008; SAS Institute Inc.).

## Results

### Subjects

A total of forty-eight subjects were evaluated. Of the subjects, fifteen were lean and thirty-three were obese. Subject characteristics can be found in Table 1. Briefly, lean subjects were 44·2 (se 2·5) years, with a BMI of 22·6 (se 0·3) kg/m^2^ and a waist circumference of 80·1 (se 2·1) cm, while obese subjects were 37·8 (se 1·7) years, with a BMI of 35·3 (se 0·8) kg/m^2^ and a waist circumference of 110·8 (se 2·0) cm. The level of total plasma BCAA in lean subjects was 1·3 (se 0·06) (×10^7^) AU *v.* 1·6 (se 0·08) (×10^7^) AU (*P* = 0·005) in obese subjects. The individual BCAA were also significantly different in lean *v.* obese subjects (isoleucine: 4·4 (se 0·3) (×10^6^) AU *v.* 5·6 (se 0·3) (×10^6^) AU, *P* = 0·006; leucine: 5·4 (se 0·2) (×10^6^) AU *v.* 6·5 (se 0·3) (×10^6^) AU, *P* = 0·02; valine: 3·1 (se 0·2) (×10^6^) AU *v.* 4·1 (se 0·2) (×10^6^) AU, *P* = 0·0006). Plasma B-AIBA levels in lean subjects were 2·5 (se 0·3) (×10^4^) AU *v.* 1·9 (se 0·2) (×10^4^) AU (*P* = 0·08) in obese subjects.

**Table 1.** Subject characteristics (*n* 48) (Mean values with their standard errors)

	Normal-weight (*n* 15)		Obese (*n* 33)		
	Mean	se	Mean	se	*P*
Age (years)	44·2	2·5	37·8	1·7	0·07
Sex (no. male)	8		13		0·28
Race (% Caucasian)	37·5		62·5		0·22
BMI (kg/m^2^)	22·6	0·3	35·3	0·8	<0·0001
Waist circumference (cm)	80·1	2·1	110·8	2·0	<0·0001
VAT (cm^2^)	53·0	13·7	123·7	10·3	<0·0001
SAT (cm^2^)	139·4	21·1	503·4	30·4	<0·0001
HOMA-IR	0·6	0·1	2·3	0·5	<0·0001
Matsuda index	15·5	1·8	7·0	1·0	<0·0001
Glucose AUC during OGTT (mmol/l×min) (×10^4^)	0·072	0·0033	0·089	0·0050	0·04
TAG (mmol/l)	0·561	0·049	1·338	0·149	<0·0001
HDL (mmol/l)	1·55	0·06	1·18	0·05	<0·0001
LDL (mmol/l)	2·57	0·21	3·02	0·15	0·09
BCAA					
Total BCAA (AU) (×10^7^)	1·3	0·06	1·6	0·08	0·005
Isoleucine (AU) (×10^6^)	4·4	0·3	5·6	0·3	0·006
Leucine (AU) (×10^6^)	5·4	0·2	6·5	0·3	0·02
Valine (AU) (×10^6^)	3·1	0·2	4·1	0·2	0·0006
B-AIBA (AU) (×10^4^)	2·5	0·3	1·9	0·2	0·08

VAT, visceral adipose tissue; SAT, subcutaneous adipose tissue; HOMA-IR, homeostasis model assessment for insulin resistance; OGTT, oral glucose tolerance test; BCAA, branched-chain amino acids; AU, arbitrary units; B-AIBA, β-aminoisobutyric acid.

### Branched-chain amino acids and β-aminoisobutyric acid

Individual and total BCAA were not associated with B-AIBA (isoleucine: *R* −0·007, *P* = 0·96; leucine: *R* −0·07, *P* = 0·67; valine: *R* −0·05, *P* = 0·74; total BCAA: *R* −0·04, *P* = 0·79).

### Branched-chain amino acids, β-aminoisobutyric acid, amino acids and measures of body composition

**Fig. 1.**
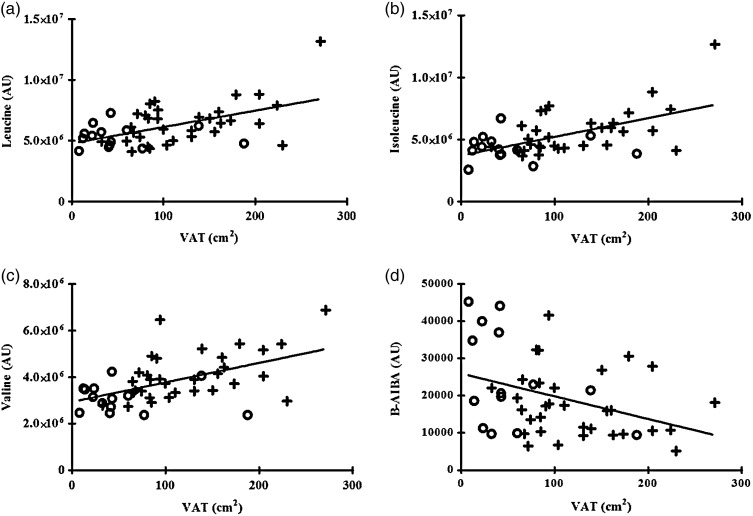
Correlation between individual plasma branched-chain amino acids and β-aminoisobutyric acid (B-AIBA) *v.* visceral adipose tissue (VAT). (a) Leucine *v.* VAT; *R* 0·44, *P* = 0·002. (b) Isoleucine *v.* VAT; *R* 0·50, *P* = 0·0004. (c) Valine *v.* VAT; *R* 0·48, *P* = 0·0007. (d) B-AIBA *v.* VAT; *R* −0·27, *P* = 0·007. ○, Lean subjects; +, obese subjects; AU, arbitrary units.

Individual and total BCAA were significantly associated with BMI (all *P* < 0·05), waist circumference (all *P* < 0·01) and VAT (all *P* < 0·005) as detailed in Table 2 and Fig. 1.

**Table 2.** Univariate regression analyses of body composition to individual branched-chain amino acids (BCAA) and β-aminoisobutyric acid (B-AIBA)

	Leucine (*R*)	Leucine (*P*)	Isoleucine (*R*)	Isoleucine (*P*)	Valine (*R*)	Valine (*P*)	BCAA (*R*)	BCAA (*P*)	B-AIBA (*R*)	B-AIBA (*P*)
BMI	0·32	0·02	0·4	0·005	0·53	0·0001*	0·42	0·003	−0·28	0·054
Waist circumference	0·41	0·004	0·5	0·0004*	0·58	<0·0001*	0·5	0·0004	−0·18	0·24
VAT (CT)	0·44	0·002	0·5	0·0004*	0·48	0·0007*	0·49	0·0006	−0·27	0·07
SAT (CT)	0·19	0·2	0·28	0·06	0·4	0·006	0·29	0·052	−0·37	0·01
Total fat % (DXA)	0·01	0·93	0·1	0·52	0·2	0·18	0·1	0·53	−0·41	0·004
Total lean % (DXA)	−0·1	0·64	−0·1	0·63	−0·21	0·15	−0·11	0·43	0·36	0·01

VAT, visceral adipose tissue; CT, computed tomography; SAT, subcutaneous adipose tissue; DXA, dual-energy X-ray absorptiometry.

* Significantly associated.

**Fig. 2.**
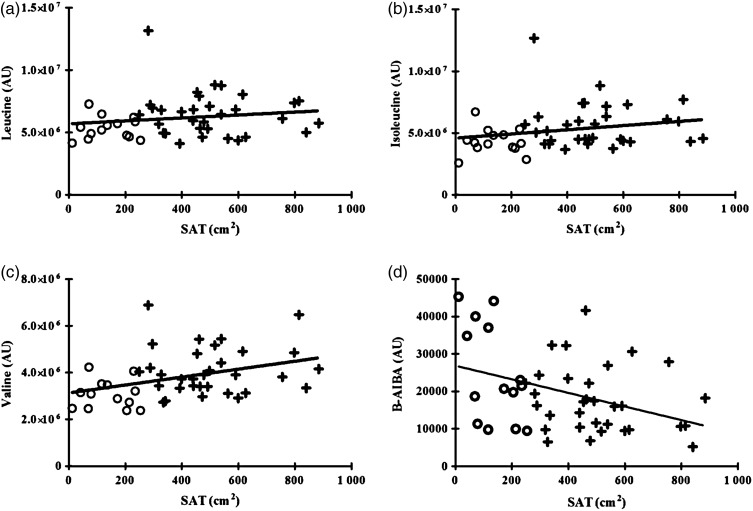
Correlation between individual plasma branched-chain amino acids and β-aminoisobutyric acid (B-AIBA) *v.* subcutaneous adipose tissue (SAT). (a) Leucine *v.* SAT; *R* 0·19, *P* = 0·20. (b) Isoleucine *v.* SAT; *R* 0·28, *P* = 0·06. (c) Valine *v.* SAT; *R* 0·40, *P* = 0·006. (d) B-AIBA *v.* SAT; *R* −0·37, *P* = 0·01. ○, Lean subjects; +, obese subjects; AU, arbitrary units.

Valine was positively associated with SAT, and isoleucine and total BCAA trended to an association with SAT, but leucine was not significantly associated with SAT (Table 2 and Fig. 2). BCAA were not significantly associated with percentage body fat and percentage lean body mass (Table 2).

Multivariate modelling correcting for age, sex and race continued to demonstrate a significant relationship between total BCAA and waist circumference (*P* = 0·0004) and VAT (*P* = 0·002), independent of the other factors. When correcting for total fat mass, age, sex and race the significant relationship between total BCAA and VAT (*P* = 0·23) was no longer significant. Likewise, in multivariate modelling correcting for BMI, age, sex and race, the relationship between total BCAA and VAT was no longer significant (*P* = 0·52). Furthermore, in multivariate modelling including both VAT and the Matsuda index VAT was no longer significant (*P* = 0·61) while the relationship between the Matsuda index and BCAA remained significant (*P* = 0·0001).

B-AIBA was significantly associated with SAT (*R* −0·37; *P* = 0·01), percentage body fat (*R* −0·41; *P* = 0·004), total fat mass (*R* −0·34; *P* = 0·02) and percentage lean body mass (*R* 0·36; *P* = 0·01), and trended to an association with BMI and VAT (both *P* < 0·10), but not waist circumference (Figs 1 and 2 and Table 2). However, none of these relationships remained significant upon multivariate modelling correcting for age, sex and race.

### Branched-chain amino acids, β-aminoisobutyric acid, amino acids and indices of insulin sensitivity

**Fig. 3.**
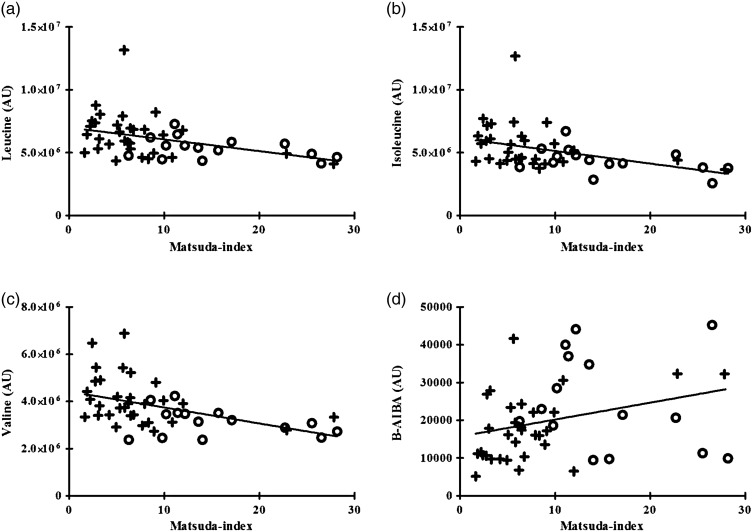
Correlation between individual plasma branched-chain amino acids and β-aminoisobutyric acid (B-AIBA) *v.* Matsuda index. (a) Leucine *v.* Matsuda index; *R* −0·44, *P* = 0·002. (b) Isoleucine *v.* Matsuda index; *R* −0·50, *P* = 0·0004. (c) Valine *v.* Matsuda index; *R* −0·53, *P* = 0·0002. (d) B-AIBA *v.* Matsuda index; *R* 0·36; *P* = 0·01. ○, Lean subjects; +, obese subjects; AU, arbitrary units.

Individual and total BCAA were significantly associated with the HOMA-IR index (all *P* < 0·005), Matsuda index (all *P* < 0·005) and glucose AUC during OGTT (all *P* < 0·005), as detailed on Table 3 and Fig. 3.

**Table 3.** Univariate regression analyses of metabolic parameters to individual branched-chain amino acids (BCAA) and β-aminoisobutyric acid (B-AIBA)

	Leucine (*R*)	Leucine (*P*)	Isoleucine (*R*)	Isoleucine (*P*)	Valine (*R*)	Valine (*P*)	BCAA (*R*)	BCAA (*P*)	B-AIBA (*R*)	B-AIBA (*P*)
HOMA-IR	0·41	0·004	0·50	0·0003	0·47	0·0008	0·48	0·0007	−0·38	0·009
Matsuda index	−0·44	0·002	−0·50	0·0004	−0·53	0·0002	−0·50	0·0004	0·36	0·01
Glucose AUC during OGTT	0·53	0·0001	0·50	0·004	0·50	0·0003	0·53	0·0001	−0·30	0·04
TAG	0·48	0·0006	0·50	0·0004	0·50	0·0003	0·51	0·0003	−0·12	0·43
HDL	−0·42	0·003	−0·38	0·009	−0·46	0·001	−0·43	0·003	0·21	0·16
LDL	0·20	0·18	0·30	0·04	0·24	0·1	0·26	0·08	−0·10	0·51

HOMA-IR, homeostasis model assessment for insulin resistance; OGTT, oral glucose tolerance test.

Multivariate modelling correcting for age, sex, race and BMI continued to demonstrate a significant relationship between total BCAA and the HOMA-IR index (*P* = 0·03). In this model, age and BMI also remained associated with BCAA (age: *P* = 0·04; BMI: *P* = 0·006). Multivariate modelling also demonstrated a significant relationship between total BCAA and the Matsuda index (*P* = 0·01). Age and BMI also remained associated with BCAA in this model (age: *P* = 0·05; BMI: *P* = 0·004). In additional multivariate modelling glucose AUC (*P* = 0·003) also remained significantly associated with BCAA independent of age, sex, race and BMI.

B-AIBA was negatively associated with the HOMA-IR index (*R* −0·38; *P* = 0·009) and glucose AUC during OGTT (*R* −0·30; *P* = 0·04) and positively associated with the Matsuda index (*R* 0·36; *P* = 0·01) (Table 3 and Fig. 3). However, these associations no longer remained significant after multivariate modelling correcting for age, sex, race and BMI.

### Branched-chain amino acids, β-aminoisobutyric acid and dietary intake

Neither plasma individual BCAA, nor total BCAA, nor B-AIBA were associated with absolute dietary protein, carbohydrate or fat intake (data not shown).

## Discussion

In the present study, we demonstrated, for the first time, a significant association between individual and total BCAA and VAT. We also confirmed the known association of BCAA with insulin resistance.

We showed, as hypothesised, a significant positive association between BCAA and VAT in this study. This suggests that adipose tissue may play an under-appreciated, but potentially significant, role in BCAA homeostasis^(^^6^^,^^19^^)^. The three BCAA, valine, leucine and isoleucine, are essential amino acids and cannot be endogenously synthesised. As the dietary intake of proteins was not related to plasma BCAA levels in this study, the plasma levels of BCAA may primarily reflect inhibited catabolism of BCAA, as well as a decreased insulin sensitivity of the skeletal muscles leading to reduced inhibition of BCAA release by the skeletal muscle^(^^7^^)^. The catabolism of BCAA begins with the transport of BCAA into the cell, initiated by branched-chain amino acid aminotransferase (BCAT) in the mitochondrion^(^^20^^)^. There are two forms of BCAT: mitochondrial (BCATm) and a cytosolic (BCATc). BCATm is found in nearly all tissues, including adipose tissue^(^^21^^,^^22^^)^. BCAT catalyses reversible transamination of BCAA to form their α-keto acids^(^^23^^,^^24^^)^. The second step of BCAA catabolism is irreversible oxidative decarboxylation, catalysed by the branched-chain α-ketoacid dehydrogenase (BCKD) complex, which is located in the mitochondrial matrix^(^^20^^,^^23^^,^^24^^)^. BCKD is the rate-limiting step in BCAA catabolism and its activity is decreased by increased acetyl-CoA concentration and NADH:NAD^+^ ratio possibly due to β-oxidation of NEFA^(^^6^^)^. Thus, increased NEFA present in obesity may decrease the activity of BCKD, thereby decreasing the catabolism of BCAA.

The importance of adipose tissue in BCAA catabolism has been demonstrated in several studies. Protein levels of BCATm and BCKDE1, one of the three catalytic components of BCKD, are reduced in *ob/ob* mice, diet-induced obese mice and Zucker fatty rats^(^^19^^,^^25^^)^. Moreover, Herman *et al*.^(^^21^^)^ demonstrated that transplanting adipose tissue from wild-type littermates to BCAT2 knock-out mice can reduce circulating BCAA^(^^21^^)^. Human studies corroborate these findings. mRNA expression of BCKDHA, the human gene that encodes for the α subunit of E1 of BCKD, was reduced in adipocytes of obese insulin-resistant human subjects compared with lean subjects^(^^19^^)^. In addition, obese women with the metabolic syndrome have lower mRNA expression of BCKD in VAT as compared with healthy obese women without disturbed glucose metabolism^(^^19^^)^. Furthermore, obese patients who have undergone gastric bypass surgery to lose weight have increased levels of both BCATm and BCKDE1a mRNA expression in adipose tissue^(^^25^^)^. These studies and our study all support a significant metabolic role of adipose tissue in BCAA homeostasis.

We demonstrated a significant association between B-AIBA and percentage lean body mass. Although lean body mass as quantified by DXA consists of all non-fat, non-bone tissue, including but not limited to striated muscle, this association is consistent with the hypothesised role of B-AIBA as a muscle-derived metabolite. We also demonstrated a significant negative association between B-AIBA and total body fat percentage and SAT, although these relationships were no longer significant upon multivariate analyses. These results suggest that increased plasma B-AIBA concentration is associated with a more favourable body composition, e.g. more lean mass and more SAT *v.* VAT.

BCAA and B-AIBA were not significantly associated with each other in our study. While the lack of association does not necessarily rule out a direct relationship between BCAA and B-AIBA, it does suggest complexity in the generation and metabolism of B-AIBA and furthermore suggests that B-AIBA is not simply a breakdown product of valine^(^^14^^,^^15^^)^.

Our results also confirmed the association between BCAA and markers of insulin sensitivity^(^^5^^–^^7^^,^^9^^–^^11^^,^^26^^)^ and lipid profile^(^^27^^)^ as previously demonstrated. The relationship of BCAA with HOMA-IR, the Matsuda index and glucose AUC after OGTT were strong and remained significant even after controlling for age, sex, race and BMI. Therefore, the role of insulin resistance in mediating the relationship between BCAA and VAT need to be considered. The addition of HOMA-IR or the Matsuda index to the multivariate regression model assessing the relationship between BCAA and VAT resulted in the loss of statistical significance between BCAA and VAT, suggesting that this relationship may be dependent upon insulin resistance.

In our study B-AIBA was not related to lipid parameters unlike a previous report^(^^14^^)^. Yet, B-AIBA did show an inverse relationship with markers of IR in our study.

Our study has some important limitations. First, we were not able to quantify the molar concentrations of BCAA and B-AIBA as no internal standard was used in the metabolite profiling. However, as the main focus of this investigation was the association between these metabolites and measures of body composition, the AU used are sufficient for that purpose and molar concentrations are not essential. Furthermore, with a relatively small sample size of forty-eight subjects, the study may not have been powered to detect true associations between BCAA, B-AIBA, and some measures of body composition or metabolism. Thus, lack of association in the present study should not be interpreted as absence of association.

The present study demonstrates an interesting relationship between BCAA and VAT. Shaham *et al*.^(^^18^^)^ previously demonstrated that BCAA responses after OGTT remained higher in subjects with an impaired glucose tolerance compared with subjects with a normal glucose tolerance. This suggests that the inhibiting effect of insulin on proteolysis can be seen after an oral glucose challenge. Future research investigating the contribution of VAT to dynamic changes in BCAA in a post-absorptive state such as after an OGTT may also be of interest. In addition, suppression of lipolysis has been suggested to be more sensitive to the actions of insulin compared with suppression of protein catabolism^(^^18^^)^. Given the possible role of NEFA in mediating BCAA catabolism^(^^6^^)^, assessing the contribution of fasting, nocturnal and post-absorptive NEFA in this context would also be warranted in future studies. Furthermore, consideration of the role of liver fat in BCAA metabolism may also be necessary in future studies given the known association between VAT and hepatic steatosis^(^^4^^)^ and the known role of the liver in amino acid metabolism.

To conclude, we demonstrate, for the first time, a significant positive relationship of plasma BCAA levels with VAT, which did not persist when adjusting for total fat mass, BMI or the Matsuda index. Furthermore, we once more showed a positive relationship of plasma BCAA and markers of insulin resistance. We also demonstrated a positive relationship of B-AIBA with lean body mass and a negative relationship with SAT and markers of insulin resistance. This study also adds further data supporting negative effects of VAT on cardiometabolic health. The results might suggest a more significant role of adipose tissue in BCAA homoeostasis than previously considered and indicate that further research in the field is needed. Furthermore, our results on B-AIBA and markers of cardiometabolic health together with other published studies^(^^14^^,^^15^^)^ suggest that further research into the regulation of this small molecule metabolite and its physiological significance is warranted.

## Supplementary material

For supplementary material accompanying this paper visit http://dx.doi.org/10.1017/jns.2015.37. click here to view supplementary material
